# AAV Vectors Vaccines Against Infectious Diseases

**DOI:** 10.3389/fimmu.2014.00005

**Published:** 2014-01-21

**Authors:** Karen Nieto, Anna Salvetti

**Affiliations:** ^1^Tumor Immunology Program (D030), German Cancer Research Center (DKFZ), Heidelberg, Germany; ^2^Centre International de Recherche en Infectiologie (CIRI), INSERM U1111, CNRS UMR5308, Ecole Normale Supérieure de Lyon, Université de Lyon, Lyon, France; ^3^LabEx Ecofect, Université de Lyon, Lyon, France

**Keywords:** AAV vectors, anti-viral vaccination, humoral responses, cytotoxic responses, antibody gene transfer, immunoadhesins, capsid

## Abstract

Since their discovery as a tool for gene transfer, vectors derived from the adeno-associated virus (AAV) have been used for gene therapy applications and attracted scientist to this field for their exceptional properties of efficiency of *in vivo* gene transfer and the level and duration of transgene expression. For many years, AAVs have been considered as low immunogenic vectors due to their ability to induce long-term expression of non-self-proteins in contrast to what has been observed with other viral vectors, such as adenovirus, for which strong immune responses against the same transgene products were documented. The perceived low immunogenicity likely explains why the use of AAV vectors for vaccination was not seriously considered before the early 2000s. Indeed, while analyses conducted using a variety of transgenes and animal species slowly changed the vision of immunological properties of AAVs, an increasing number of studies were also performed in the field of vaccination. Even if the comparison with other modes of vaccination was not systemically performed, the analyses conducted so far in the field of active immunotherapy strongly suggest that AAVs possess some interesting features to be used as tools to produce an efficient and sustained antibody response. In addition, recent studies also highlighted the potential of AAVs for passive immunotherapy. This review summarizes the main studies conducted to evaluate the potential of AAV vectors for vaccination against infectious agents and discusses their advantages and drawbacks. Altogether, the variety of studies conducted in this field contributes to the understanding of the immunological properties of this versatile virus and to the definition of its possible future applications.

## Introduction

Historically, vaccination strategies against infectious agents have mostly used live attenuated pathogens. These vaccines are highly efficient for generating both humoral and cellular immune responses but for many pathogens this approach is too risky even in their attenuated form to be used in humans. Subunit vaccines, usually recombinant proteins, have provided an interesting and safe alternative; however their use is limited to the generation of antibody (Ab) responses and is, therefore, limited to preventive vaccination strategies against pathogens that can be efficiently cleared by a humoral response. In addition, their efficiency frequently requires repeated injections of high doses of the vaccines coupled to adjuvants. In this context, the development of viral vector has provided a very interesting alternative since they can efficiently deliver antigens (Ag) into the antigen processing pathway leading to the stimulation of cytotoxic T cell responses, which are essential to clear intra-cellular pathogens and to develop therapeutic vaccines (1).

Viral vectors are derived from wild type viruses by deleting a part or all of viral genes. The immunogenic properties of a viral vector results not only from that of Ag which is expressed, but also on the intrinsic biological properties of the viral particle which determine its interaction with the cells of the immune system, in particular antigen presenting cells (APC), and with other target tissues. Both contribute to the nature and the potency of the immune response that is induced (1). So far, the most widely evaluated viral vectors for vaccination, in particular, in human clinical trials are those derived from adenovirus (Ad) and the poxvirus family (2). Both of these types of vectors provide several advantages as vaccines because of their efficiency of infection of several cell types including APC. However, when used as a vaccine both types of vectors contain, in addition to the transgene encoding for the Ag, several viral genes whose expression can constitute a safety concern and lower or modify the efficacy of the vaccine by diverting immune responses from the Ag itself. In addition, for both vector types, if the strong immunogenicity of the viral particle itself may be seen as helping to induce strong immune responses, it can also constitute a safety problem, due to the strong inflammatory responses that are induced. Last but not least, as for many other viral vectors, a main issue is that of the pre-existing immunity in the human population, which may constitute a barrier to their use in man.

Adeno-associated virus (AAV) vectors already have a relatively long history in gene therapy but have only recently emerged in the vaccination field. Since the first and unique report by Manning et al. (3) documenting the capacity of AAV to induce a strong humoral and cellular response against the herpes simplex virus (HSV) type 1 glycoprotein B (3), an increasing number of studies have explored the use of AAV vectors for genetic vaccination, thus contributing to the understanding of their immunological properties. This review is focused on the description of vaccination studies conducted mostly against viral or microbial antigens and using AAV vectors directly *in vivo* to highlight their properties, potential limitations, and future developments. Neither the few studies which used AAV vectors for vaccination against non-infectious diseases nor the use of these vectors for immunotherapy by *ex vivo* gene transfer into dendritic cells (DC) are included. The two first sections summarize the main characteristics of AAV vectors when used in various vaccination settings. The third section presents the results from the most advanced studies, which explored the potential of AAV vaccines against experimental challenge in a relevant animal model and/or have explored the efficacy of AAV-mediated vaccination in non-human primates (NHP). Finally, the last part of this review describes the most likely future developments in this field.

## AAV Vectors for Active Immunotherapy

Compared to other viruses used as vectors for vaccination and in particular to Ad and poxviruses, AAV potentially offers a significant number of advantages. First, the vectors are derived from a non-pathogenic virus that is inherently replication defective (4). Accordingly, several preclinical and clinical gene therapy trials have demonstrated their favorable safety profile (5, 6). The vectors are gutless and, therefore do no encode for any viral gene. The vector genome is usually composed of a single-stranded (ss) DNA molecule containing the transgene expression cassette flanked by the viral inverted terminal repeats [for a review, see Ref. (7)]. AAV particles containing a double-stranded, also called self-complementary (sc) AAV genome, can be also developed to improve the kinetics and the level of expression of the transgene (8). AAV vectors possess the capacity to efficiently transduce several tissues *in vivo* and the isolation of several AAV serotypes and of a multitude of capsid variants potentially offers the possibility to develop prime/boost strategies by switching the AAV capsid, thus avoiding the anti-capsid neutralizing humoral responses induced after the first injection. However, as with other viral vector systems, AAVs also have a number of drawbacks, notably the limited transgene capacity, a strong and wide pre-existing immunity in humans, and the technological challenge of producing large and high titter vector stocks. The studies conducted in the field of active vaccination using AAV vectors are very diverse in terms of targets, objectives, and strategies (Table 1). However, so far, only a limited number of studies have been conducted directly comparing AAV vectors to other vector vaccines. Despite this diversity and lack of comparative studies, several common conclusions can be extrapolated from these studies which define the advantages and also the pitfalls of AAV vectors for this particular application.

**Table 1:** **Summary of active immunization studies using AAV vectors**.

Antigen	AAV serotype	Injection route	Reference
HSV-1 (gB and gD)	AAV2	IM/mice	Manning et al. (3)
HPV (E7, E7/hsp70 L1)	AAV2	IM/mice	Liu et al. (9)
	AAV2	IM/mice	Liu et al. (10)
	AAV5	IN/mice	Kuck et al. (11)
	AAV5, 8, 9	IN/mice	Nieto et al. (12)
	AAV1, 2	IM/mice	Zhou et al. (13)
	AAV5, 9	IN/macaques	Nieto et al. (14)
HIV (env, tat, rev)	AAV2	IM, SC, IN, IP/mice	Xin et al. (15)
	AAV2	Oral/mice	Xin et al. (16)
	AAV1, 3, 4, 5, 7, 8	IM/mice	Xin et al. (17)
	AAV1, 2, 5, 7, 8, 9	IM/mice	Lin et al. (18), Lin et al. (19)
	AAV8, rh32-33	IM/mice	Lin et al. (20)
	scAAV2, 7, 8	IM/mice	Wu et al. (21)
SIV	AAV2	IM/macaque	Johnson et al. (22)
SARS-CoV (S protein)	AAV2	IM/mice	Du et al. (23)
Malaria (MSP4, 4/5)	AAV1, 3	IM/mice	Logan et al. (24)
Influenza (NP, H1, M1)	AAV8, rh32.33	IM/mice	Lin et al. (20)
	AAV9	IM/mice	Sipo et al. (25)
DEV (Env)	AAV8, rh32.33	IM/mice	Li et al. (26)
TB (Ag85A)	Modified AAV2	IM/mice	Rybniker et al. (27)
NIV (G protein)	AAV1, 8, rh32.33	IM, ID/mice	Ploquin et al. (28)

*IM, intra-muscular; IN, intra-nasal; ID, intra-dermal; IP, intra-portal; IPL, intra-pleural; HSV-1, herpes simplex virus type 1; HPV, human papillomavirus; HIV, human immunodeficiency virus; SIV, simian immunodeficiency virus; SARS-CoV, severe acquired respiratory syndrome coronavirus; DEV, dengue virus; TB, Mycobacterium tuberculosis, NIV, Nipah virus*.

### Evaluation of different AAV serotypes and routes of immunization

Initial analyses conducted in the field of vaccination have been performed using AAV2-derived vectors. Despite their lower efficiency compared to other AAV serotypes (29), in these initial studies, AAV2 vectors already demonstrated their capacity to induce strong immune responses using a variety of injection routes and viral Ag derived from HSV, the human immunodeficiency virus (HIV), the severe acquired respiratory syndrome coronavirus (SARS-CoV), and the human papillomavirus (HPV) (3, 10, 15, 16, 30) (Table 1). Notably, a study using AAV2 vectors expressing several simian immunodeficiency virus (SIV) genes indicated that a single intra-muscular administration of the vector was able to elicit SIV-specific T cells and Ab, demonstrating its potential in a macaque NHP model (22). Thereafter, other AAV serotypes rapidly entered the field and out-performed AAV2 in terms of immune responses, as expected from their higher efficiency for gene transfer (13, 17–19, 28). The use of various AAV serotypes also allowed varying the injection routes (Table 1). In particular, several studies have used AAV5 or AAV9 vectors for intra-nasal vaccination to induce a mucosal immunity (11, 12). Most notably, nasal application of AAV9 was efficient even in the presence of high levels of pre-existing serum anti-AAV9 neutralizing antibodies (NAb), which did not prevent re-immunization with the same serotype and transgene expression (14, 31).

### Induction of humoral responses

A remarkable feature of AAV vector vaccines in most, if not all studies, is their capacity to induce strong and long lasting Ab responses, even after a single administration. Several studies documented the induction of humoral responses lasting for many months and sometimes more than 1 year (3, 11, 12, 26, 28). In addition, in some studies, the AAV-induced Ab response was higher and more sustained than using other vaccination strategies including DNA, recombinant proteins, inactivated virus, or virus-like particles (VLPs) (3, 10–12, 24, 30). Such pronounced Ab response may be linked to the high and sustained expression of the transgene over time achieved with most AAV serotypes (18, 19, 26, 28). The high and long lasting level of Ab responses may also explain why, in some instances, a boost effect was not always observed upon re-injection with the same or an alternative AAV serotype (22, 24, 28). In contrast, some studies have documented the possibility of enhancing the AAV-induced humoral response by using an Ad vector expressing the same Ag for the boost (18, 19). These observations suggest that the enhancement of humoral responses can be achieved only after stimulation of different immune pathways than those used for the initial prime vaccination. This scenario is similar to that described for vaccination strategies using plasmid DNA for the prime injection and an heterologous vaccine for the boost (1).

### Induction of CD8^+^ T-cell responses

The control of several infectious diseases requires, in addition to a strong humoral response, the concomitant induction of cytotoxic cellular responses to not only prevent virus spreading but also eradicate virus-infected cells. Since the first report using AAV for vaccination, several studies have documented the induction of transgene-specific CD8^+^ T-cell responses following injection of AAV vectors in mice (3, 12, 13, 15, 17, 25) and NHP (22). However, only two studies have thoroughly analyzed such cellular responses quantitatively and qualitatively. Two reports, published in 2007 indicated that CD8^+^ T-cell responses induced by AAV vectors, derived from a variety of natural AAV serotypes, failed to be successfully recalled and amplified upon a boost with an Ad vector indicating a default in the CD8^+^ T-cell memory response. A detailed analyses of such responses further showed that CD8^+^ T cells had markers of exhaustion, which were correlated to a continuous expression of the transgene (18, 19). Whether the persistence of transgene expression was a consequence or a cause of such a functionally impaired CD8^+^ T-cell response is still unclear. Interestingly however, a functional transgene-specific CD8^+^ T-cell response could be induced by changing the AAV capsid. A hybrid capsid AAVrh32.33, derived from two natural rhesus macaque isolates, was able to generate a CD8^+^ T-cell response against the transgene product in mice and NHP which could be successfully amplified following a boost with an Ad vector (20). Further studies conducted in mice demonstrated that intra-muscular administration of the AAVrh32.33 vector was able to induce a strong cellular response even against transgene products, such as LacZ, which are usually tolerated using natural AAV serotypes and resulted in the elimination of AAV-transduced cells within 2 months after AAV administration (32). Altogether, these analyses indicated that the capsid, in addition to the transgene, is a key modulator of immune responses in particular by changing the tropism, and thus the interaction of AAV particles with immune cells. It is worth noting that although natural AAV serotypes are considered to be unable to efficiently transduce APC, in particular DC, it is currently unknown how AAVrh32.33 interacts with these cells. Finally, it is important to highlight that such strong differences among AAV capsids, observed in murine models may not hold true in other animals species, in particular primates, in which even natural AAV vectors were shown to induce immune responses leading to the elimination of transgene-expressing cells (33).

## AAV Vectors for Passive Immunotherapy

The ability of AAV vectors to efficiently express various transgenes including those coding for soluble proteins has fostered their use for Ab-gene transfer to produce neutralizing Ab (NAb) directly *in vivo* (Table 2). Indeed, Ab-based therapies are costly and limited by the half-life of the Ab, with single administrations resulting only in short term protection. Therefore, most of these therapies require frequent administration of relatively high doses of the Ab, often via intravenous administration, since high and persistent serum levels of Ab are frequently required for optimal clinical efficacy. In this scenario, the use of AAV vectors may be of great interest, in particular, to allow a sustained and continuous expression of the Ab after a single administration. In these studies, as in gene therapy, AAV vectors are used only as vehicles to produce high levels of proteins *in vivo* and, in contrast to the previous situation (active immunotherapy), immune responses against the transgene product, here the Ab, are unwanted. Most of the studies performed in this area are recent and have used natural AAV serotypes other than AAV2 (Table 2).

**Table 2:** **Summary of passive immunization studies using AAV vectors**.

Antigen	AAV serotype	Injection route	Reference
HIV	AAV2	IM/mice	Lewis et al. (34)
	AAV8		Balazs et al. (35)
RSV	AAVrh10	IPL/mice	Skaricic et al. (36)
SIV	AAV1, scAAV1	IM/macaque	Johnson et al. (37)
Influenza	AAV8	IM/mice, ferrets	Balazs et al. (38)
	AAV9	IN/mice, ferrets, macaques	Limberis et al. (39)

*RSV, respiratory syncytial virus. See the legend of Table 1 for additional abbreviations*.

One of the challenges in the engineering of an AAV vector coding for a full length Ab was to ensure an efficient and equimolar production of the light and heavy chains. A major advance was achieved by demonstrating the possibility to produce high levels of Ab in mice after intra-portal administration of an AAV8 vector expressing heavy and light chains linked with a 2A self-processing peptide derived from the foot-and-mouth disease virus (FMDV2A) (40). With this technology, 1 mg/ml of Ab was detected in the serum of the animals several months after AAV administration, a level much superior to that previously observed using an AAV2 vector expressing the two chains using a dual promoter system (34). This initial report fostered a series of studies to validate this strategy in various experimental models. Notably, by using specific AAV serotypes, it was possible to address Ab secretion into tissues such as the lungs, which are the site of entry of several viruses and bacteria. For example, intra-pleural administration of AAVrh10 encoding for a murine Ab against the respiratory syncytial virus (RSV) resulted in the long-term production of anti-RSV NAb in the serum and the lungs and partially protected the animals against a challenge with the virus (36). A similar strategy was developed to express *in vivo* Ab able to protect against the deleterious effects induced by some toxins or compounds such as anthrax, cocaine, or nicotine (41–43).

## Protection Studies and Pre-Clinical Evaluations

The use of AAV as vehicle for gene delivery to induce immune responses against foreign and self-antigens has been explored in animal models, but only one phase I clinical trial was performed in humans (44). For prophylactic vaccination against infectious agents, there are safety concerns since typically such vaccines are given to healthy children or adolescents with an unknown risk for late consequences. Also, prophylactic vaccines are often targeted to large populations. From this standpoint, AAV vectors are interesting tools since their use for gene therapy has already demonstrated their overall absence of toxicity (6). However, current very high costs for manufacturing AAV vectors and the need for high particle doses are clearly major hurdles for vaccine development. Nevertheless, future studies may help define some niches in which AAV may be particularly advantageous over other vaccination strategies.

This chapter will describe a number of informative pre-clinical vaccination studies, which demonstrated a complete protection against experimental challenge in a relevant animal model and/or have explored the efficiency of AAV-mediated vaccination in NHP.

### Henipaviruses

Nipah virus (NiV) and Hendra virus (HeV) are closely related, recently emerged paramyxoviruses, belonging to the henipavirus genus. Both viruses are capable of causing considerable morbidity and mortality in a number of mammalian species, including humans (45). Infection of humans is characterized by a rapid and extensive spread of the virus in several organs with symptoms including respiratory distress and encephalitis (46). These symptoms can be reproduced by experimental infection of several animal models including hamsters (47). Because of their high pathogenicity in humans, their broad tropism and the absence of any vaccine or treatment, henipaviruses are presently classified as biosafety level 4 (BSL4) agents and considered as potential biothreats (48).

The major vaccination strategy to prevent henipavirus infection has focused on direct administration of soluble forms of the G viral glycoprotein to induce a protective immune response (49–53). This form of vaccination requires several injections of the recombinant protein coupled to adjuvants to achieve a significant immune response. In a recent study, Ploquin et al. evaluated the efficiency of AAV vectors expressing the NiV G protein to induce a protective immune response (28). The evaluation of two routes of vaccination and different prime/boost strategies employing three AAV serotypes indicated that a single IM AAV injection in mice was sufficient to induce a potent and long lasting Ab response consisting of IgG and NAb. Further translational studies in hamsters demonstrated that a single injection of an AAV vector encoding NiV G was sufficient to protect 100% of the animals against a lethal challenge with NiV and 50% of the animals against a challenge with HeV, thus indicating the induction of cross-neutralizing immune responses. Altogether, this study presented a new vaccination approach whereby a single immunization is sufficient for the induction of a protective immunity against henipaviruses and opened new perspectives toward the evaluation of AAV vectors as a vaccine against these emergent infectious diseases.

### Human immunodeficiency virus

Controlling the epidemic of HIV infection worldwide remains a major challenge. Indeed, even though significant success in controlling the disease has been achieved by understanding the virus biology and by developing targeted drugs, vaccine-based preventive measures are still needed. Major challenges toward this goal are the antigenic variability and multitude of virus strains (54). Several viral vectors have been tested as vaccines against HIV, most notably poxviruses and Ad, but have met little success in protective efficacy and even adverse effects in terms of protection against HIV infection (1). This was, in particular, the case of the STEP human trial which used Ad vectors encoding HIV proteins (55).

Among several vaccine strategies, AAV vectors have been evaluated in several animal studies (Table 1). In an early study, an AAV vector expressing the HIV env, tat, and rev genes was given to BALB/c mice in single applications and by different administration routes including intramuscular and intra-nasal. This resulted in the production of a high level of HIV-specific serum IgG and fecal secretory IgA as well as in the appearance of a cytotoxic T-cell response (15). Non-invasive oral administration of AAV2 vectors expressing the HIV *env* gene was further studied by Xin and colleagues (16) who showed the induction of systemic and mucosal humoral and cellular immune response that partially protected against rectal challenge with a recombinant vaccinia virus expressing the HIV *env* gene. The most advanced studies in the field were conducted by Johnson et al. (22), who investigated the efficacy of AAV2 vectors expressing several SIV genes (rev-gag, rev-env, and RT-IN) injected intramuscularly against a challenge with SIV (22). In this study, the authors demonstrated the induction of robust T cell and Ab responses after a single vaccination. Upon challenge with SIV, complete protection was observed when low doses of SIV were given intravenously. However, only partial protection was observed at higher doses of SIV. Of note, one phase I trial with AAV2 HIVgag-protease-ΔRT demonstrated safety but modest immunogenicity (Gag-specific T cells in 16% of the recipients) (44).

A much more interesting perspective in the field was provided by two recent studies which used AAV vectors for passive immunotherapy (Table 2). This concept was first validated by Johnson and colleagues, who used an AAV1 vector to express imunoadhesins (IA) against SIV in macaques (37). IA are chimeric Ab-like molecules that are generally composed of the Fc region of an immunoglobulin linked to the ligand-binding region of a receptor or adhesion molecule. IA offer the advantage of being small molecules whose sequence can be easily accommodated in an AAV vector and eventually in a scAAV vector, which packages a double-stranded DNA genome enhancing the kinetics and level of transgene expression (8). Johnson and colleagues demonstrated that intra-muscular injection of the scAAV1 vector resulted in a higher level of secretion of the IA than using a conventional ss AAV1 and generated a long lasting neutralizing activity in the serum. Importantly, most vaccinated animals were protected against infection with SIV and all were protected against the disease. However, Ab against the IA were generated in some animals and correlated with partial protection. The potential of AAV-based passive immunotherapy against HIV infection was further demonstrated by Balazs and colleagues, who reported the lifelong expression in mice of monoclonal Ab against HIV after a single intra-muscular injection of an AAV8 vector. Importantly, humanized mice vaccinated with AAV were protected against intravenous injection of replication competent HIV at a very high dose (35).

### Human papillomavirus

Two VLPs-based vaccines are commercially available against HPV 16 and HPV 18, which are the most important risk factors for the development of cervical cancer and other malignant tumors of the anogenital tract and of the head and neck. Clinical trials and first reports after launching vaccination campaigns demonstrated highly efficient protection against persistent infection and precancerous lesions besides excellent safety profiles (56–58) [for review, see Ref. (59)]. Early data on the influence of this vaccination strategy on cancer incidence are expected to arise 15–20 years after initiation of mass immunization. Despite the high costs of the available products, in the near future, an AAV-based HPV vaccine is unlikely to become a serious contender to the existing vaccination scheme, which involves three IM injections of adjuvanted VLPs manufactured by recombinant expression of the HPV major structural protein (L1). Yet, studies with HPV 16 L1 AAVs have yielded interesting results in mice and NHP and make these vectors an interesting option for future developments.

For example, Kuck and colleagues demonstrated a sustained humoral and cellular immune response (>1 year) in mice immunized with a single intra-nasal dose of AAV5-HPV16L1. The responses by far outlasted the ones obtained after three doses of HPV 16 VLPs that had been applied by the same route. Lyophilized and re-dissolved AAV particles remained immunogenic albeit at reduced efficiency (11). Thus, AAV has the potential for a needleless vaccine and – unlike the available vaccines – does not need refrigeration since it can be stored as lyophilized powder. Both features are important in developing countries where cervical cancer is a major public health challenge. Intra-nasal immunization with AAV9-HPV16L1 was also tested in NHP (macaques). L1-specific NAb were elicited and persisted for at least 7 months post AAV administration. As expected from previous studies, the presence of pre-existing high titer AAV9 Ab did not prevent immunization with the same serotype when administered intra-nasally (14, 31).

As mentioned before, the introduction of prophylactic HPV vaccination has the potential to significantly reduce the worldwide burden of HPV-related disease. However, even in countries with sufficient resources there will always be individuals that, for reasons of ignorance about HPV as a human carcinogen or active objection against vaccination *per se*, will not benefit from such programs. Therefore, there is a medical need for the development of HPV-specific therapeutic vaccines against an established infection. Such strategies involve cytotoxic T cells against early proteins that are expressed in persistently infected and HPV-transformed cells [for a review, see Ref. (60)]. One of the intensively studied targets, namely the viral oncoprotein E7 has also been analyzed as the transgene in AAV vectors (Table 1). Two studies used AAV vectors coding for a 19 aa HPV 16 E7 peptide, which contains a well-defined mouse (H-2b)-restricted CTL epitope or the complete HPV 16 E7 gene fused to the *Mycobacterium tuberculosis* heat shock protein 70 (hsp70) (9, 13). A single intra-muscular immunization of C57/BL6 mice induced E7-specific CD4^+^ and CD8^+^ T lymphocytes and – upon challenge with syngeneic E7-transformed cells – a significant delay or a complete inhibition of tumor growth. In experiments aiming toward an HPV 16 vaccine that combines prophylactic and therapeutic properties, Nieto and colleagues analyzed different AAV serotypes that carried a fusion gene of L1 and the major part of E7 (12) by a single intra-nasal immunization of mice. The AAV5 and the AAV9 (not the AAV8) vectors efficiently induced both humoral and cellular immune responses that were superior to vaccination with HPV16-L1 VLPs or HPV16-L1/E7 chimeric VLPs. In addition, vaccination with the AAV vectors led to a significant protection of animals against a challenge with different HPV tumor cell lines.

### Influenza virus

The currently available vaccines are insufficient to keep in check the seasonal influenza outbreaks that affect people of all ages and claim at least one millions of lives in children up to 5 years of age worldwide (61). The reason vaccines are relatively less successful is the high variability of the virus and highly type-specific nature of the vaccines that need to be designed for the actual emerging virus strain. The unavoidable delay between the identification of a new variant and availability of the appropriate vaccine – particularly owing to the laborious manufacturing process of virus replication in chicken eggs – leaves the population unprotected at least against the first wave of a new epidemic.

Earlier studies have demonstrated strain-specific immunization and protection against lethal challenge. Sipo and colleagues generated AAV9 vectors expressing the hemagglutinin (HA), nucleoprotein (NP), or matrix protein (M1) genes of the A/Mexico/4603/2009 (H1N1) isolate, a pandemic influenza of swine origin. After the single injection of a mixture of two or three AAV serotypes, they obtained complete protection against a homologous challenge and partial protection against a heterologous and highly virulent strain (25). The authors argued that, although this vaccine candidate will not induce sterilizing immunity it may mitigate the clinical symptoms, diminish the transmission rate, and, thus, generate a herd immunity before the homologous classical vaccines have been made available. A slightly more recent study also examined the protection level in mice vaccinated with AAV vectors expressing NP. In particular, the objective of those studies was to evaluate the efficacy of protection in mice receiving high doses of pooled human IgG to mimic a situation in which a pre-existing immunity against AAV may interfere with the vaccine. Interestingly in those studies, the authors showed that an AAVrh32.33 vector-expressing NP could fully protect the animal against a challenge with lethal doses of influenza virus strain PR8 (20). Altogether, these studies demonstrated the efficiency of strain-specific AAV vaccines.

However, as for other RNA viruses, vaccination against seasonal influenza outbreaks is complicated by the continuous emergence of new variants or strains which escape NAb generated by the previous infection. The recent isolation of cross-neutralizing Ab, which bind to a conserved region of HA has recently allowed the evaluation of new vaccination strategies based on passive immunotherapy and aimed at generating a long-term broadly protective humoral activity *in vivo*. Interestingly, two independent groups simultaneously reported the use of AAV vectors to produce, *in vivo*, either a full length human Ab against HA or an IA directed against the same protein (38, 39). In the first study, the authors injected the AAV8 vector intramuscularly in mice and ferrets and demonstrated that the long-term production of the anti-HA Ab in the serum conferred complete protection against five different influenza strains (38). In the second study, an AAV9 vector was administered intranasally to induce production of the IA in the nose and the lungs (39). An advantage of this mode of delivery is that vector expression is localized to the nasal epithelia and is not expected to be widely disseminated in the body. In addition, the natural turnover of the airway epithelial cells may ensure that the vector is not permanently present *in vivo*. As in the first study, the animals were protected against the IN challenge with different influenza strains. Importantly, in this latter example, the authors also showed that the time between AAV injection and challenge could be reduced to 3 days, demonstrating the potency of this expression system (39). However, for both studies, analyses performed in larger animal species, in particular ferrets, resulted in only partial or no protection probably because of the emergence of an immune response against the human Ab or the IA. Of course, an immune response against the human Ab is not expected in humans. Whether the same holds true for IA is still unclear. Even though some important issues, such as the potential immunogenicity of IA and the translation of this approach in larger animals still need improvements, these approaches clearly indicate that AAV vectors are powerful tools for passive immunotherapy which certainly deserve further studies in NHP models.

## Conclusion and Future Directions

The studies conducted so far have highlighted several important advantages of AAV vectors for vaccination and notably, in the case of active immunotherapy, their capacity to induce sustained levels of Ab responses after a single injection or in passive immunotherapy, as tools for secreting Ab directly into the circulation. These features are linked to the capacity of AAV vectors to persist for long periods in the transduced tissues and clearly distinguish AAV from other viral vectors, such as Ad and poxviruses, with which a rapid elimination of transduced cells is observed *in vivo*. Despite these properties, many drawbacks still hamper the use of these vectors as a vaccine in humans. This last section will review the major problems, which remain to be solved and discuss possible solutions.

### Improving AAV immunogenicity

The rationale for using AAV vectors for genetic vaccination is mainly based on their intrinsic absence of pathogenicity, their capacity to infect a variety of tissues, and to express transgenes at a high and sustained level. When using AAVs for passive immunotherapy, these properties are sufficient to consider AAV vectors as very promising tools even though safety studies are required to evaluate the effects of a continuous secretion of Ab *in vivo*. However, in the case of active immunotherapy, these properties may not always be sufficient to ensure an efficient vaccination. Indeed, even if shown capable to induce transgene-specific immune responses in large animal models (33) and to activate innate responses at modest but detectable levels (62), AAV vectors are still considered to possess a low immunogenic profile, compared to other viral vectors, in particular Ad vectors. This is notably illustrated by the persistence of transgene expression observed in several vaccination studies and by the reported lack of functional CD8^+^ T-cell responses observed with natural AAV serotypes (19, 20, 28). This aspect clearly represents a major disadvantage for using AAVs for preventive or therapeutic vaccination trials requiring the induction of robust cytotoxic T-cell responses. Therefore, the use of these vectors for these applications requires enhancement of their intrinsic immunogenic properties. Several studies already indicate that this is possible, notably through the manipulation of the viral genome and the capsid.

#### Increasing AAV immunogenicity by manipulating the vector backbone

Adeno-associated virus vectors can be composed of a ss or a sc DNA genome. Changing the nature of the DNA genome has a profound impact on the kinetics and the level of transgene expression by bypassing the need for DNA second-strand synthesis before transcription of the vector cassette (8). Accordingly, the comparison of ss and scAAV vectors for passive immunotherapy indicated that the latter produced higher levels of Ab than the former (37). Interestingly, several recent studies indicated that this modification could also impact on the immunogenic properties of the vectors. Indeed, modifying the vector backbone enhanced both innate and transgene-specific adaptive immune responses (21, 63). In particular, using an AAV vector expressing a secreted version of the HIV Gag protein, Wu and colleagues showed that scAAV vectors of different serotypes induced more potent CD8^+^ T-cell and Ab responses than conventional ssAAV (21). However, as previously observed with conventional ssAAV vectors a progressive loss of function of CD8^+^ T cell was observed, indicating that the modification of the nature of the vector genome was not sufficient to generate fully functional T-cell responses. In addition, this strategy was applicable only to transgenes that could be accommodated in an expression cassette which was one half that of conventional AAV vectors.

#### Increasing AAV immunogenicity by manipulating the capsid

Another strategy to enhance transgene-specific immune responses induced by AAVs consists of changing the viral capsid. Obviously, the level and the nature of the immune response are tightly linked to the nature of the capsids, which determines the tropism of the particle and the efficiency of transduction. Accordingly, several studies documented significant differences in the levels of immune response induced by natural AAV serotypes (12, 18, 19, 28). Presently, more than a 100 AAV variants have been isolated from human and non-human tissues and approximately 13 are used to produce vectors (29). An even greater variety is offered by the possibility of genetically modifying the capsid either by creating artificial variants or by inserting specific immunogenic peptides on the capsid surface as discussed below (64).

Regarding the generation of new artificial AAV variants, the most compelling evidence was provided by studies performed with the AAVrh32.33. This variant is an engineered hybrid between two natural AAV rhesus macaque isolates, which was specifically selected for its immunogenic properties. The analysis of the immune response induced by this variant indicated that it was able to induce a vibrant and functional CD8^+^ T-cell response directed against the Ag, unlike the other natural AAV serotypes, and exploiting this capacity was beneficial for vaccine development (20, 32). The recent structural analysis of this variant further indicated that the functional T-cell activating domain lies within the VP3 portion of the capsid (65). A future deeper understanding of the immunological properties of the capsid domains of AAV may then lead to the rational design of artificial variants capable of differentially stimulating immune responses.

Other interesting strategies to increase AAV immunogenicity have focused on the generation of capsid exposing selected epitopes. AAV particles are composed of 60 capsid protein subunits, named VP1, VP2, and VP3 (66). Efficient methods to generate genetically modified capsid exposing selected peptides have been developed in the recent years (67). Due to the highly structured and repetitive presentation of epitopes on the capsid, potent B-cell responses against this peptide are expected. Two recent studies illustrate the feasibility of this approach. Nieto and colleagues inserted two neutralizing epitopes from the L2 protein of HPV16 and HPV31 into two different positions of VP3 and used assembled empty AAV particles – AAVLP(HPV16/31L2) – to vaccinate mice and rabbits. AAVLP (HPV16/31L2) empty particles coupled to montanide adjuvant, induced high levels of Ab able to cross neutralize several HPV types (68). In a similar approach, Rybniker and colleagues used genetically modified AAV2 capsids displaying at their surface Ag85A, a well described Ag from *M. tuberculosis*. Using such modified capsids to package a vector over-expressing the same Ag, increased the kinetics of Ab production as well as their avidity, compared with an AAV assembled into a wild type capsid. In addition, insertion of the antigen at the capsid surface was also shown to be sufficient to induce a memory B-cell recall response (27).

Both of these strategies illustrate the potential of capsid modification for manipulating the immune response and it is likely that, in a very near future, new AAV vectors specifically selected for their ability to induce strong transgene-specific immune responses will continue to emerge.

### Circumventing anti-AAV pre-existing immunity

Epidemiological studies indicate that anti-AAV Ab can be detected in the majority of the population worldwide and that their seroprevalence varies according to the AAV serotype and the geographical region (69–71) [for a review, see Ref. (72)]. As a result, the efficacy of AAV vectors for *in vivo* gene transfer can be severely reduced. In animal models, the use of an alternative AAV serotype is sufficient to circumvent anti-AAV NAb induced by previous immunization with a different capsid. However, this strategy may not be valid in humans in which potentially wider cross-reacting immune responses exist. Interestingly however, artificial variants such as AAVrh32.33 showed a much lower seroprevalence than natural serotypes, with less than 2% of the population testing positive worldwide, indicating that it could represent a good candidate for vaccination (69). An additional strategy to circumvent pre-existing immunity is provided by the possibility to engineer AAV capsids with mutations targeting key immunogenic amino acids. This sophisticated strategy was developed by Maersch and colleagues, who demonstrated its feasibility by using the *in vitro* directed evolution method to select AAV particles capable of escaping anti-AAV2 NAb (73).

### Reducing AAV vector doses

In gene therapy trials, the use of AAV vectors in humans requires very high vector doses in order to achieve a therapeutic efficiency. For example, doses of approximately 10^12^ vector particles per kilogram were required in the initial Hemophilia B trials using AAV2 vectors (5). Even if lower doses were used in the latter trials using other AAV serotypes, the amount of particles delivered as a single injection in patients still remained impressively high. Experiments performed in animals for vaccination with AAVs also used very high doses to achieve an efficient immune response. Therefore, as for gene therapy, application of high vector doses may constitute a safety concern by increasing the risk of inducing detrimental immune responses and of off-target transduction. Hence, future efforts to reduce vector doses should focus on decreasing the vector load by improving transduction efficiencies and increasing the immunogenicity of the vector particles.

## Conflict of Interest Statement

The authors declare that the research was conducted in the absence of any commercial or financial relationships that could be construed as a potential conflict of interest.
